# First report on cutaneous infectious granuloma caused by *Schizophyllum commune*

**DOI:** 10.1186/s12879-018-3187-5

**Published:** 2018-06-26

**Authors:** Lidi Tian, Yunzhu Mu, Hao Zhang, Xiaocui Su, Chuan Yang, Xianzhu Shu, Defu Qing

**Affiliations:** 1 Department of Dermatology, Affiliated Hospital of North Sichuan Medical College, Nanchong, China; 2 Department of Dermatology, Shenzhen Baoan Central Hospital and the 5th Affiliated Hospital of Shenzhen University Health Science Center, Shenzhen, China

**Keywords:** Cutaneous granulomas, Itraconazole, *Schizophyllum commune*

## Abstract

**Background:**

*Schizophyllum commune*, a basidiomycetous fungus, is a common invader of rotten wood. This fungus rarely causes mycotic disease in humans, especially cutaneous infection. In this paper, we describe the first case of cutaneous granuloma caused by *S. commune* in a Chinese woman.

**Case presentation:**

A 25-year-old female with a two-year history of erythema, papules, nodules, and scales on her sole of left foot was presented to our outpatient center. Samples were obtained by the scraping of lesion and for light microscopy. Hyphae were observed by microscopic examination. We carried out a skin tissue biopsy, which showed multiple granulomatous nodules. Biopsy specimens were also inoculated onto media. After being cultured on SDA at 27 °C for 7 days, spreading-woolly-white colonies grew on the inoculation sites of media containing chloramphenicol only and there^,^s no other colonies grew. *S. commune* was identified by morphology methods, biochemical tests, and PCR sequencing. Pathological findings also aided in diagnosing cutaneous fungal granuloma. Oral itraconazole was applied. After 1 month of therapy, rashes on her left foot and pain were improved.

**Conclusion:**

We describe the first case of cutaneous granuloma caused by *Schizophyllum commune,* which illustrates the importance of recognizing uncommon pathogenic fungal infections.

## Background

*Schizophyllum commune* is a common fungal species, which belongs to Eukaryota, Opisthokonta, Fungi, Dikarya, Basidiomycota, Agaricomycotina, Agaricomycetes, Agaricomycetidae, Agaricales, Schizophyllaceae, Schizophyllum. This fungus colonizes diverse trees and rotting woods worldwide [1–3]. *S. commune* has also long been regarded as nonpathogenic to humans [4, 5]. Recently, an increasing number of cases related to *S. commune* infection has been reported. Most reports are associated with allergic bronchopulmonary mycosis (ABPM) after inhaling the spores of *S. commune* [1, 2, 6]. Infection in deep tissues, such as the sphenoid sinus, maxillary sinus, and brain, has also been recently reported [2, 7–9]. To our knowledge, the cutaneous infection caused by *S. commune* has not been reported yet.

## Case presentation

**Fig. 1:**
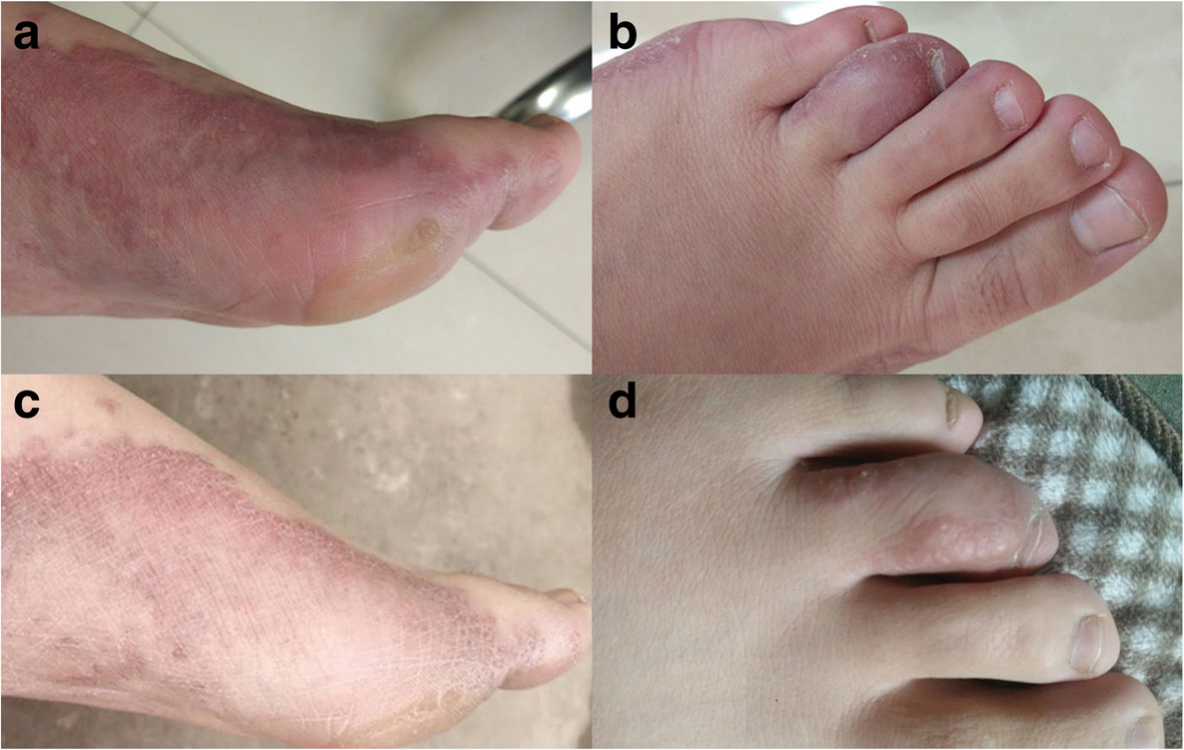
**(a, b)** Sole of left foot exhibits erythema, papules, nodules, and scales. The fourth toe dorsum is also infected. **(c, d)** Foot lesions regressed after 1 month of treatment

A 25-year-old female with a two-year history of erythema, papules, nodules, and scales on her sole of left foot was presented to our outpatient center. She has no history of autoimmue disease and untreated with immunosuppressive therapy. Considering her pregnancy, she was not given treatments for 1 year. The left foot skin lesion on the medial and lateral margins and on the fourth toe dorsum became enlarged with evident pain after more than 1 year (Fig. 1a and b). Approximately 1 month before visiting our department, she received treatment ineffectively in a local clinic, and the diagnosis was unclear.

**Fig. 2:**
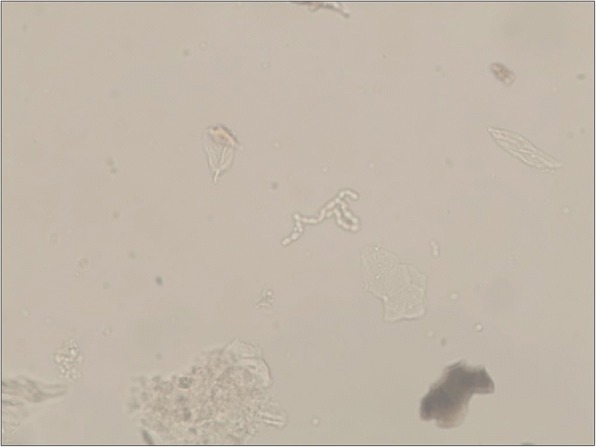
Hyphae were observed by microscopic examination (400×)

Samples were obtained by the scraping of lesion and for light microscopy. Hyphae were observed by microscopic examination (Fig. 2).

**Fig. 3:**
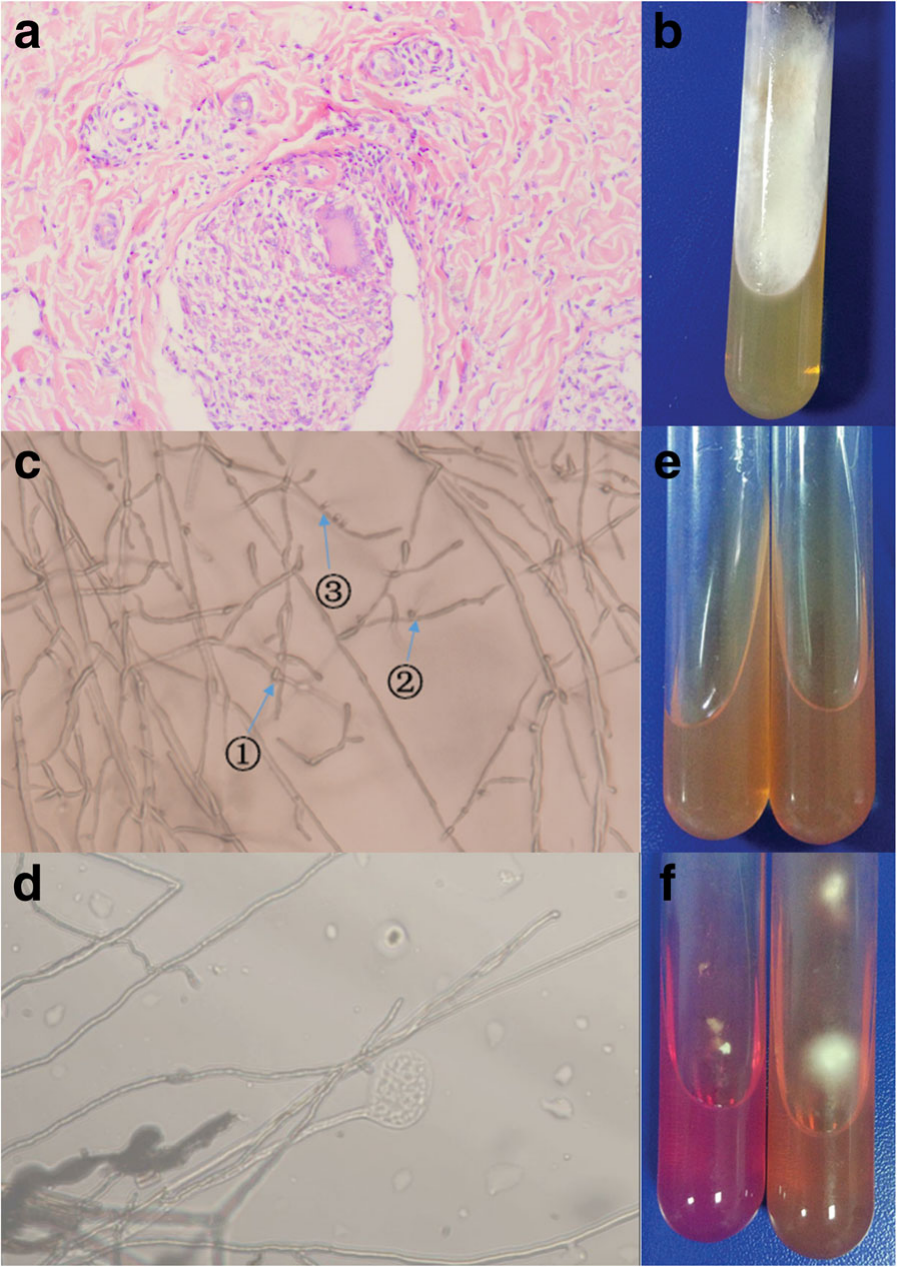
**a** Hematoxylin-eosin (H&E) stain for pathology study showed multiple granulomatous nodules (× 200). **b** When cultured on Sabouraud^’^s dextrose agar, spreading-woolly-white colonies grew on the media containing chloramphenicol only. **c, d** Clamp connections (①)**,** spicules (②), tear-like secretions(③), and medusa-like isomers were observed on the slide culture at 27 °C after 3 days (400×). **e, f** Urease activity test before and after cultivation: isolated strain (+), *Trichophyton rubrum* standard strain (−)

We carried out a skin tissue biopsy, which showed multiple granulomatous nodules (Fig. 3a). The Ziehl–Neelsen stain was negative. Periodic acid–Schiff (PAS) and Grocott methenamine silver (GMS) staining were carried out two times. Results were also negative. Biopsy specimens were also inoculated onto two kinds of media: Sabouraud’s dextrose agar (SDA), where one of which contained chloramphenicol and cycloheximide, and the other one contained chloramphenicol only. After being cultured on SDA at 27 °C for 7 days, spreading-woolly-white colonies grew on the inoculation sites of media containing chloramphenicol only and there^,^s no other colonies grew (Fig. 3b). The colonies produced an unpleasant smell like biogas. No colony was observed on the media with chloramphenicol and cycloheximide. Clamp connections, spicules, tear-like secretions, and medusa-like isomers were observed on the slide culture at 27 °C after 3 days (Fig. 3c and d). Urease activity tests were also performed. *Trichophyton rubrum* standard strain and the isolated strain were cultured on urease media at 27 °C for 7 days. The *T. rubrum* standard strain was negative, whereas the isolated strain turned red (Fig. 3e and f).

Sequencing of large subunit rDNA was performed by using the E.Z.N.A.™ Fungal DNA Mini Kit (Omega Biotek, USA). We utilized set primers for the region of internal transcribed spacer (ITS) and performed PCR. The PCR primers were ITS_1_: 5′-TCCGTAGGTGAACCTGCGG-3′ and ITS_4_: 5′-TCCTCCGCTTATTGATATGC-3′. The PCR-amplified DNA was matched with that of *S. commune* (Nos. KP 326677.1 and KP 004975.1) with a homology of 100%. After identification, the sequence was submitted to the GenBank (MF 495704).

Pathological finding and mycological examination indicated a cutaneous granuloma caused by *S. commune*. Oral itraconazole (100 mg) was applied twice a day. The rashes on the left foot and the pain regressed after 1 month of treatment (Fig. 1c and d). Follow-up is currently under way.

## Discussion and conclusions

To our knowledge, fungal granuloma is caused by a wide variety of fungi, such as *Coccidioides*, *Histoplasma*, *Blastomyces*, *Cryptococcus*, *T. rubrum*, and *T*. *mentagraphytes*, but some less known fungi have been recently documented as causes of fungal granuloma [10]. In the past, *S. commune* was regarded as a rare human pathogen. Since 1950 when Kligman reported the first case of onychomycosis caused by *S. commune*, this fungus has emerged as an increasingly important pathogen for clinical disease [11]. We conducted a literature review through PubMed and found that *S. commune* is an opportunistic pathogenic fungus that can cause sinusitis and ABPM mostly [3, 12], as well as rarely isolated from patients with onychomycosis, palate ulceration [11, 13]. Table 1 shows the clinical spectrum of fungal disease caused by *S. commune* and country-wise distribution of clinical cases. Cutaneous and subcutaneous infections caused by *S. commune* have not been previously reported.

**Table 1:** Clinical spectrum of disease due to Schizophyllum commune and country-wise distribution of clinical cases (*n* = 99)

Mycosis (No. of case)	Country (No. of cases)	References
Sinusitis (34)	Austria (5)	[14, 15]
	USA(3)	[8, 16]
	Colombia(3)	[17–19]
	France (3)	[20–22]
	Serbia (2)	[23, 24]
	India(2)	[25, 26]
	U.K. (1)	[27]
	Japan (7)	[28–30]
	Australia (1)	[31]
	New Zealand(2)	[5, 32]
	South Korea(3)	[2, 33]
	China(2)	[34, 35]
ABPM (30)	Japan(29)	[3, 5, 36–49]
	India(1)^a^	[50]
Bronchial mucoid impaction (8)	Japan(8)	[38, 45, 51–56]
Pulmonary fungal ball (2)	North America(1)	[57]
	India(1)	[50]
Schizophyllum asthma(2)	Japan(2)	[58]
Pulmonary infiltrate plus eosinophilia (1)	Japan(1)	[38]
Chronic eosinophilic pneumonia (1)	Japan(1)	[59]
Honeycomb lung (1)	Japan(1)	[60]
Bronchogenous cyst (1)	Serbia (1)	[61]
Pulmonary nodules (1)	Taiwan (1)	[62]
Other pulmonary mycoses (11)	Iran (7)	[63]
	Japan (3)	[38]
	Italy (1)	[64]
Brain abscess (2)	USA (1)	[9]
	Austria (1)	[15]
Ulceration of the palate (1)	Colombia (1)	[13]
Otitis externa (1)	Slovenia (1)	[65]
Fatal Empyema Thoracis (1)	Hong Kong (1)	[66]
Onychomycosis (1)	USA (1)	[11]
Meningitis (1)	Brazil (1)	[67]

^a^include only one case, but the patient has the allergic broncho-pulmonary mycosis and bronchial mucoid impaction at the same time

In our case, colonies grew on the inoculation sites of media with no other colonies grew and antifungal therapy only was effective, which can be excluded the possibility of contamination. We describe the first case of cutaneous granuloma caused by *S. commune,* which illustrates the importance of recognizing uncommon pathogenic fungal infections.

## Abbreviations

ABPM: Allergic bronchopulmonary mycosis
GMS: Grocott methenamine silver
ITS: Internal transcribed spacer
PAS: Periodic acid–Schiff
PCR: Polymerase chain reaction
*S. commune*: *Schizophyllum commune*
SDA: Sabouraud^’^s dextrose agar,
*T. mentagraphytes*: *Trichophyton mentagraphytes*
*T. rubrum*: *Trichophyton rubrum*
